# A Snapshot of Transdermal and Topical Drug Delivery Research in Canada

**DOI:** 10.3390/pharmaceutics11060256

**Published:** 2019-06-01

**Authors:** Mahdi Roohnikan, Elise Laszlo, Samuel Babity, Davide Brambilla

**Affiliations:** Faculty of Pharmacy, University of Montreal, Montreal, QC H3T 1J4, Canada; mahdi.roohnikan@umontreal.ca (M.R.); elise.laszlo@umontreal.ca (E.L.); samuel.babity@umontreal.ca (S.B.)

**Keywords:** transdermal drug delivery, Canada, skin, permeation enhancers

## Abstract

The minimally- or non-invasive delivery of therapeutic agents through the skin has several advantages compared to other delivery routes and plays an important role in medical care routines. The development and refinement of new technologies is leading to a drastic expansion of the arsenal of drugs that can benefit from this delivery strategy and is further intensifying its impact in medicine. Within Canada, as well, a few research groups have worked on the development of state-of-the-art transdermal delivery technologies. Within this short review, we aim to provide a critical overview of the development of these technologies in the Canadian environment.

## 1. Introduction

The skin is the most accessible organ of the body, stretching over a surface area of 1.7 m^2^ and making up roughly 10–16% of the total mass of the body [1,2]. Its primary function is to act as a protective layer against environmental hazards such as chemicals, heat, and toxins, as well as to defend the body against invading microorganisms and allergens. Moreover, the skin plays a key role in homeostasis and body temperature regulation, and acts as a sensory organ exposed to the environment, detecting external stimuli such as temperature, pressure, and pain [2].

While the skin might appear to be an ideal route of administration for local and systemic therapeutics, it actually represents a challenging barrier against the penetration of most compounds [3]. It is composed of three main layers—the epidermis, the dermis, and the hypodermis—with a total thickness ranging from 0.5 to 4 mm, dictated by various factors such as body region, age, and sex [1,3]. The epidermis is a non-vascularized, multilayered stratum whose cells receive nutrients via diffusion from the lower layers, with the outermost portion, the stratum corneum, acting as the main barrier against the passage of drugs [4]. The stratum corneum has a wall-like structure with corneocytes—non-nucleated keratinocytes composed of 70–80% keratin and 20% lipids—acting as “bricks” in a network of intercellular lipids, while desmosomes act as structural links between the “bricks” [5]. Beneath the epidermis is the dermis, which contains a network of blood vessels, which provide nutrients, regulate body temperature, and remove waste products; as well as a network of lymphatic vessels, which are important in regulating interstitial pressure and clearing large molecules [5]. These networks are critical for the distribution of molecules crossing the epidermis into the systemic circulation. When a skin-permeable chemical is applied to the surface of the skin, this process creates a concentration gradient between the surface and the dermis which helps to drive the drug into the skin over time. Thus, the capillary network embedded in the dermis is the main target for transdermal delivery strategies. Finally, the innermost layer, the hypodermis, is mostly composed of adipose tissue and its primary roles are to provide thermal insulation, protect against physical shock, and serve as an energy reserve.

Though transdermal and topical drug delivery has important advantages, including bypassing the harsh conditions of the gastrointestinal tract, as well as the pain and requirement for trained personnel associated with parenteral administration, the skin represents a significant physical barrier and only a very limited number of drugs are compatible with this route of administration without the use of permeation promoters. An important effort has thus been focused on the design of more efficient strategies to facilitate the permeation of drugs across the skin, while avoiding tissue damage. These strategies have drastically improved small molecule delivery for cosmetic, dermatological, and other localized applications, and have allowed the delivery of macromolecules and vaccines in clinical trials, along with a few other systemic applications [6,7,8].

The industrial sector for topical and transdermal drug delivery has grown as the global market continues to prosper. In addition to established transdermal formulations developed by larger manufacturing companies, there are a number of initiatives focusing on new systems, and numerous start-ups have been successfully seeded in this expanding market [9]. The general acceptance of transdermal products by patients is very high, which is also evident in their increasing market share, worth $20.5 billion in 2010 and currently estimated at over $32 billion [9,10]. Canada is the world’s 10th largest pharmaceutical market with a 1.9% share of the global market, and annual domestic pharmaceutical manufacturing was valued at $9.6 billion in 2017 [11]. Hence, research on topical and transdermal drug delivery systems is of great importance in Canada. In this brief review, we present and discuss the works of Canadian research groups in the field, critically comparing them to similar research performed outside the country, as well as highlighting instances where research has translated to the private sector. We categorized the proposed systems based on their nominal nature and/or performance mechanisms, namely: chemically enhanced, physically enhanced, and nanoparticle-based delivery systems (Table 1). We believe that this short review suitably fits the overall topic of this Special Issue, entitled “Drug Delivery Technology Development in Canada”, and will be useful for the community in positioning Canada within this important research field.

## 2. Chemical Permeation Enhancer-Based Systems

Chemical permeation enhancers (CPEs)—generally defined as molecular compounds able to destabilize the stratum corneum and facilitate the passage of drugs while, ideally, limiting or avoiding deeper tissue damage—have long been used in transdermal and topical formulations [12]. In the past, several compounds, such as alcohols, surfactants, terpenes, and fatty acids, have been shown to enhance the permeation of therapeutics, however, only a few have been adopted in commercial transdermal and topical products [13,14].

A remarkable example of the use of CPEs to help deliver larger molecules across the stratum corneum has been proposed by a group at the University of Waterloo. The system, initially described in a patent in 1998 [15], consists of multilamellar biphasic lipid vesicles (BPVs) ranging in size from 0.1–10 µm. These vesicles were proposed to contain a lipophilic dispersion within an aqueous core, surrounded by over 15 concentric phospholipid bilayers separated by additional aqueous phases. This allows molecules of interest (otherwise too large to cross the stratum corneum) to be loaded throughout the vesicle, enabling local or systemic delivery, with permeation enhanced by the incorporation of solvents and surfactants within the vesicle structure. Importantly, the works demonstrated that the relative proportions of lipids and permeation enhancers used in these formulations can vary significantly, possibly as a function of the molecule being delivered. Two of the most abundant enhancers, namely propylene glycol and oleic acid, were included in a study of the mechanisms of permeation enhancement by CPEs [16]. Though both were defined as solvents, oleic acid was demonstrated to increase fluidity and disorder in the structural lipids of the stratum corneum, whereas propylene glycol was speculated to primarily enhance permeation through solubilization of the molecule being delivered. Early uses of this technology were described in the context of the transdermal delivery of insulin and vaccines [17,18], highlighting the initial promise of the delivery method, though neither application has thus far led to clinical or commercial translation. Nonetheless, research into this topic appears to be ongoing, with more recent articles describing mechanistic studies of vesicle delivery and the use of BPVs for the topical (rather than transdermal) delivery of interferon (IFN)-α in the context of human papilloma virus (HPV) treatment. It was demonstrated in a guinea pig model that, when applied topically, the treatment effectively delivered IFN-α to the dermis, reaching a maximum local concentration of ~100,000 IU/100 cm^2^ within 6 h, and remaining at a therapeutically effective level for up to 72 h [19]. However, more comprehensive studies of the half-life of IFN-α in the skin were not conducted. Additionally, when compared to an intradermal injection (the standard delivery method for IFN-α), the vesicle-based topical application never generated elevated systemic concentrations (<100 IU/mL), while the intradermal administration resulted in rapid systemic absorption of the drug and consequently an increased likelihood of adverse side-effects. Overall, dermal application of BPV-IFN-α led to a sustained local release of IFN-α with minimal systemic exposure [19]. The authors reported that the topical IFN-α was generally well tolerated by animals, as no significant difference in body weight, apparent pain, or visual appearance of the skin was observed between animals treated with the vesicle-based IFN-α, those treated with a placebo formulation, and animals that did not receive treatment. In cases where irritation or redness did occur (3.5%), it was minor and resolved itself within 2 h of application. The transdermal delivery mechanism of IFN-α was investigated using confocal microscopy, differential scanning calorimetry (DSC), and small- and wide-angle X-ray scattering (SAXS/WAXS) [20]. Confocal microscopy analysis indicated that encapsulated IFN-α was delivered across the stratum corneum to the viable epidermis and dermis, at a depth of roughly 70 µm. Data extracted from DSC and SAXS/WAXS analyses suggest that a three-dimensional cubic Pn3m polymeric phase rearrangement of intercellular lipids is induced by the interaction between stratum corneum lipids and the biphasic vesicles, possibly explaining the improved delivery of IFN-α. More importantly, in another work, the authors investigated the delivery of BPV-IFN-α in humans [21]. The particles were demonstrated to be between 1000 and 1100 nm in size and their zeta potentials were measured between 70 and 78 mV. Following the application of 5, 15, and 40 MIU/g formulations of encapsulated IFN-α to the upper, inner arm of healthy volunteers as a topical patch, dermal levels of IFN-α were measured to be 120 ± 30, 380 ± 60, and 400 ± 80 IU/mg respectively, suggesting a limit to the local concentration deliverable through this system. These local concentrations indicated a delivery of between 3% and 5% of the dose contained in the patch to the skin, comparable to many topical formulations of small molecules [22]. In a pilot study of 12 patients with external Condylomata acuminata warts (a topical manifestation of HPV infection in the genitals) the application of a significantly lower dose of encapsulated IFN-α (1 MIU/g) twice daily for two weeks resulted in a decrease in lesion size and 2’,5’-oligoadenylate synthetase activity (a marker for viral infection), as well as a significant decrease in systemic HPV viral load [21]. In light of this demonstrated potential for IFN-α delivery, multilamellar BPVs could be envisioned as suitable candidates for the non-invasive topical delivery of other therapeutic macromolecules to the skin. Despite these promising initial results, the most recent publication concerning these biphasic vesicles was released in 2013, likely owing to the acquisition of the technology by the Vancouver-based company Altum Pharmaceuticals Inc. Branded as BiPhasix™, Altum appears to have further developed the technology in the context of IFN-α delivery, and has conducted clinical trials of a vesicle-containing cream for treatment of HPV in females, with Phase III trials slated to begin in Q2 2019, according to their website [23,24]. While the specific use of multilamellar BPVs for topical or transdermal delivery was quite unique (likely owing to the proprietary nature of the technology), no other examples of work on similar lipid-based transdermal delivery systems were found in Canada. For the simplest forms of lipid-based carriers (namely single-layer liposomes), this can be attributed to their limited success at breaching the stratum corneum without additional permeation enhancers, and ambiguity regarding their mechanism of transdermal delivery [25]. Despite this, over the past few years there has been a growing interest in the concept of nanostructured lipid carriers (NLCs) as a means of overcoming some of the difficulties associated with traditional liposomes [26]. Similar to some of the principles used in the design of multilamellar biphasic vesicles, NLCs consist of a solid lipid nanoparticle with a variable percentage of liquid lipids and surfactants included within, resulting in a disordered internal structure, and allowing increased drug loading and improved skin permeation. While many groups have been studying this delivery route [27,28,29], none are based in Canada, highlighting a potential topic of interest for groups studying topical or transdermal delivery. In particular, since the systemic delivery of insulin, vaccines, and other larger therapeutic compounds through BPVs never reached clinical stages, NLCs could present another avenue for investigation, especially as they have been primarily studied in the context of small molecule delivery.

Another important class of CPEs is ionic liquids (ILs); low melting point salts that have drawn interest for their uses in green chemistry, but are also being investigated based on their potential for transdermal drug delivery [30,31,32]. Indeed, ILs have shown the ability to facilitate transdermal transport, bypassing the physical barrier of the stratum corneum through disruption of cellular structure, lipid bilayer fluidization, and generation of permeation routes, all of which facilitate the diffusion of drugs to the dermis [33,34,35,36]. For instance, Zakrewsky et al. have demonstrated that choline-based ILs can enhance the transdermal delivery of mannitol, a small hydrophilic molecule with low skin permeability [35]. Importantly, this interest in ILs has also extended to the design of ILs based on active pharmaceutical ingredients (API-ILs); the combination of an API with an optimal counterion results in a new chemical entity with improved pharmaceutical properties, including better solubility and ADME characteristics [37]. Although ionized forms of a drug typically cross biological membranes to a lesser extent (owing to their charged nature), limiting their transport through lipid membranes, recent reports have demonstrated that additional interactions (e.g., hydrogen bonding) with a counterion can promote their co-transport to a greater extent than the free ionic drug [38,39,40,41,42]. However, despite the advantages they offer, research into the transdermal applications of API-ILs remains in early stages. Recently, a group at McGill University described the development of ILs to improve the delivery of poorly soluble drugs through the skin. Zavgorodnya et al. studied the effects of various counterions on the membrane permeability of salicylate-based APIs-ILs [43]. Specifically, they paired three counterions (choline, tributylammonium, and triethylene glycol monomethyl ether tributylammonium) with a salicylate anion to generate three different API-ILs and assessed their impact on transdermal diffusion using a silicone membrane as a skin mimic. Remarkably, each of the ILs showed increased transmembrane diffusion relative to sodium salicylate dissolved in triethylene glycol monomethyl ether, suggesting that the counterions play an important role in the permeation of salicylate, beyond simple solubilization. In particular, the polyethylene glycol (PEG)-functionalized counterion (triethylene glycol monomethyl ether tributylammonium) enhanced transdermal transport up to 2.5-fold relative to the non-PEGylated tributylammonium. The improved permeation observed with the PEGylated counterion was potentially due to capacity of triethylene glycol to act as a CPE while also improving the dissolution of the whole IL complex [44,45]. Although further evaluations, both ex vivo and in vivo, are needed to confirm the actual potentials of these salicylate formulations, the same group proceeded to evaluate the in vivo transdermal delivery of lidocaine—a common local anesthetic often selected as a model compound due to its limited transdermal permeability—using a similar strategy [46,47]. For this study, the authors prepared two API-IL pairs: lidocainium docusate ([Lid][Doc]) and Lidocaine·Ibuprofen (Lid·Ibu)—which have previously been reported to generate strong hydrogen bonds, promoting transport across a synthetic membrane [39]—and compared them to lidocainium chloride ([Lid]Cl). To perform in the in vivo tests, each form was dissolved in a commercially-available moisturizing vehicle cream (LUBRIDERM^®^), topically applied to shaved rats, and the plasma profile of concentration vs. time was assessed by ELISA assay. Among the different forms, Lid·Ibu demonstrated the greatest and most rapid systemic exposure of lidocaine (4 h AUC of 12, 602, and 1763 µM·h for [Lid][Doc], [Lid]Cl, and Lid·Ibu). Interestingly, the [Lid][Doc] API-IL displayed a drastically lower plasma concentration compared to the salt API ([Lid]Cl), which could be due to the strong hydrophobicity of the ionic salt and the high molecular weight of the counterion. Importantly, after application of Lid·Ibu, the authors also measured the plasma profile of ibuprofen and observed a higher plasma concentration relative to lidocaine, in contrast with previous observations in synthetic membranes, where the two drugs showed the same kinetics [47].

This phenomenon requires more investigation; a possible explanation could be that the two compounds permeate the stratum corneum in paired form, and become dissociated within the complex skin matrix, leading to different plasma absorption kinetics. Despite the relative novelty of the API-IL strategy for transdermal and topical delivery, early works appear to indicate that it is a promising method for the enhancement of transdermal and topical delivery, which could be applied to a vast range of low molecular-weight drugs [47,48]. Nonetheless, more systematic studies are needed, to investigate different counterions and their effects on drug absorption, cytotoxicity, and skin irritation in order to better understand their mechanism of action, and to compare them with other transdermal and topical delivery systems.

## 3. Physical Permeation Enhancer-Based Systems

Physical permeation enhancers use a physical process to promote the passage of drugs through the superficial layers of the skin, avoiding damage to the deeper layers. While some of these methods permit the delivery of drugs across the stratum corneum without damage—for instance iontophoresis, which uses an electrical field to promote the electrophoretic mobility of a drug—others cause only superficial physical disruption to the skin. Indeed, one conceptually simple and effective way to bypass the stratum corneum without the use of chemical compounds is to physically pierce the superficial layers of the skin and inject the active compound. However, classical hypodermic needles are usually too large to do this without damaging the deeper layers, potentially causing pain and tissue damage. Thus, microneedles (MNs), pointed microstructures with a submillimeter length, have been developed as an alternative technique to deliver vaccines and drugs. Their potential clinical use presents the substantial advantage of being painless (as the MNs are not long enough to reach skin nociceptors) and could potentially be self-administered, similar to other topical formulations [49]. Designs for transdermal drug delivery include solid, dissolving, and hollow MNs, which can be arranged as in-plane or out-of-plane arrays. Among these, hollow MNs have the primary advantage of being able to deliver large doses (comparable to hypodermic needles) directly to the dermis and can be used with any drug without the need for optimization of the formulation, or post-manufacturing sterilization. Although different methods have been proposed for the manufacture of hollow MNs, including femtosecond laser two-photon polymerization [50] and microinjection molding [51], their commercial use has been curbed by their high costs of fabrication [52]. One important player in this field is the Stoeber group, located at the University of British Columbia in Vancouver. They introduced a new, allegedly more cost-effective process based on solvent casting, to manufacture hollow out-of-plane clay-reinforced polyimide MNs with lengths up to 250 μm [53]. To do so, they used photolithography to manufacture re-usable micromolds containing pillar-shaped MNs using an epoxy-based photoresist. The mold was then spin-coated with polydimethylsiloxane and treated with O_2_ plasma to improve its surface wetting. Using these molds, they optimized the manufacture of hollow MNs by casting a montmorillonite nanoclay powder mixed with *N*-methyl-2-pyrrolidone (NMP) dispersed in a solution of polyimide PI-2611 (85–95% *N*-methyl-2-pyrrolidone, 10–20% S-biphenyldianhydride/p-phenylendiamine) onto the mold structures. Following a 2 h evaporation of NMP at 65 °C, 250 μm MNs were formed and removed from the molds. The tips of the MNs were then opened at an aperture of 50 μm using 3 μm aluminium oxide polishing film. Mechanical tests indicated that the MNs were robust enough to penetrate rabbit skin (an in vivo model generally recognized as a suitable mimic of the thicknesses and elasticity of human skin) and efficiently deliver a 0.0025 wt % suspension of 0.21 μm fluorescent polystyrene beads when attached to a standard syringe. The same group adjusted the manufacturing process to allow the preparation of metallic MNs with the same hollow, out-of-plane geometry [54]. To do so, they used a MN mold coated with a layer of poly(methyl methacrylate) seeded with carbon black (a conductive polymer) as a cathode, and a pure nickel anode, both immersed in an electroplating solution consisting of nickel sulfate, nickel chloride, and boric acid. After the application of a 2 mA current for 150 min, a 70 μm thick nickel backing layer was obtained.

Following this electroplating process, the tips of the MNs were opened using O_2_/CF_4_ plasma etching. Subsequently, the outer surface of the MNs was covered with a 20 nm thick gold layer to avoid any dermal allergic reaction that could be caused by the nickel. Mechanical compression tests indicated that the MNs were strong enough to pierce human skin without breaking, with a measured fracture force of 4.2 ± 0.61 N. Moreover, the delivery of 2.28 μm fluorescent beads into pig skin was demonstrated, using 500 μm hollow metallic MNs with a tip lumen diameter of 40 μm. The significant advantage of this new hollow out-of-plane MN preparation process is that MNs with a wide range of heights, spacing, and lumen sizes could be prepared. Importantly, this group has funded the start-up company Microdermics^®^, and has begun clinical testing, with the primary goal of evaluating the safety of these MNs in humans [55]. Their aim was to evaluate the biocompatibility and inertness of gold- and silver-coated MNs, relative to uncoated MNs when applied to the skin, as nickel is known to cause skin irritation. Though this clinical trial was completed in 2015, no results have yet been disclosed.

## 4. Nanoparticle-Based Systems

The last decade has witnessed a remarkable rise of nanomaterials in drug delivery research, which has also translated into promising results in the field of transdermal delivery [56]. Indeed, micro- and nano-carriers are among the most sought-after methods that have been extensively studied as potential delivery systems for the transport of non-permeable molecules across the stratum corneum [57]. Specifically, microemulsions are thermodynamically stable colloidal systems containing oil and water, stabilized by a combination of surfactants and co-surfactants, that have attracted significant attention for topical and transdermal delivery purposes [58]. These systems have been studied and developed for the delivery of a vast range of compounds to and across the skin for dermatological, cosmetic, and systemic applications. In comparison with conventional emulsions, microemulsions have been claimed to enhance skin delivery primarily by virtue of their reduced droplet size and the disruption of the stratum corneum by their constituents [58]. For instance, microemulsion-based formulations for lidocaine delivery generally have a longer-lasting effect than emulsion-based ones and result in 1.5–2 times greater permeation of lidocaine than the emulsion-based EMLA^®^ cream [59,60].

At the University of Toronto, the Acosta group has investigated the design and optimization of microemulsion-based systems for transdermal drug delivery applications [61]. To do this, they used a donor-skin-receiver mass balance model to study the effects of the concentration of surfactant used to generate the microemulsions on the transdermal delivery of lidocaine. Among different classes of components, lecithin-based microemulsions have attracted attention thanks to the generally recognized as safe status of their main constituent [62]. However, lecithin (a mixture of amphiphilic substances) tends to form lamellar and other liquid-crystal phases, and the addition of co-surfactants—generally medium-chain alcohols (e.g., butanol and pentanol)—is thus necessary for the formation of stable microemulsions [63].

While presence of these co-surfactants results in the low interfacial tension and small particle size observed in the emulsions [63], they are known to have skin-irritation properties [64]. To solve this problem, the group investigated other classes of additives as co-surfactants, which led to the selection of sorbitan monooleate (Span 80) as a lipophilic linker, and a mixture of caprylic acid (CA) and sodium caprylate (SC) as a hydrophilic linker for the fabrication of stable oil-in-water (type I), water-in-oil (type II), and bicontinuous (type III or IV) microemulsions (classified by studying their phase behavior), based on an isopropyl myristate oil phase [64,65]. The ratio of Span 80 to lecithin was kept constant at 3:1, while the ratio of CA to lecithin was maintained at 0.75:1. Using these emulsions, transdermal delivery performance was assessed as a function of lecithin concentration. The droplet size (radius) was measured by dynamic light scattering and was found to be constant at 6 nm regardless of surfactant concentration. It was shown that in lecithin-linker microemulsions, an increase in surfactant concentration was associated with an increased quantity of lidocaine delivered across porcine ear skin using a MatTek permeation device [61,66]. In addition, the authors demonstrated the superior lidocaine delivery of their lecithin-linker-based formulations relative to a pentanol-based formulation, with the type II microemulsions being the most effective, with a flux of up to 0.4 mg/(cm^2^·h) compared to the maximum of 0.12 mg/(cm^2^·h) for type I microemulsions.

In vitro cytotoxicity studies using a (3-(4,5-dimethylthiazol-2-yl)-2,5-diphenyltetrazolium bromide) tetrazolium cell viability assay on human reconstructed skin showed that these lecithin-linker microemulsions had a reduced toxicity profile compared to medium-chain alcohol-based microemulsions [64]. Despite having been extensively explored, the actual mechanism by which nano- and micro-formulations can promote the delivery of compounds through the epidermis remains controversial [57]. To explain the observed permeation results with their optimized formulations, the group proposed a dominant transport mechanism: due to their small size, the microemulsion droplets migrate to the lower epidermis and upper dermis, creating a depot-like effect and release the drug into the deeper layers of the skin. However, the observed increase in permeation as a function of surfactant concentration might suggest a combined mechanism in which the surfactants destabilize the lipid structure within the stratum corneum (acting in a CPE-like manner)*,* leading to the diffusion of the nano-droplets into the deeper layers. Given this possible mechanism of action, this technology could also be considered as a CPE-based system.

It should be noted that the proposed system is likely to require further investigation. Indeed, it has been shown that when a lecithin-related, naturally-derived monoacyl phosphatidycholine (MAPL) surfactant was used, crystal-like structures formed at the surface of the skin, acting as an additional barrier and further limiting drug diffusion. This observation, combined with the limited permeation enhancement of lecithin surfactants, might raise concerns regarding the overall efficacy of this strategy for the topical or transdermal delivery of drugs [67].

Regardless of the mechanism, after establishing the transdermal delivery potential of their lecithin-based microemulsion system, the same group went on to tackle a classical problem associated with topical formulations, namely that the low viscosity of microemulsions makes them challenging to apply and localize on a designated area of skin [65,66,67,68]. As a result, a longer-releasing formula using gelatin was developed to enhance the viscosity of the system. The selected formulation containing 20% gelatin had a zero-shear viscosity close to 3 Pa·s, an order of magnitude higher than the original microemulsions and within the range of commercial topical creams (1–10 Pa·s) [69]. The authors reported that their microemulsion-based gels (MBGs) performed similarly to their lecithin microemulsions, though with a slightly lower loading and release of lidocaine.

To make these lecithin-linker microemulsions, the authors modified their previous formulation, replacing the hydrophilic linkers sodium octanoate and octanoic acid with a milder combination of PEG-6-caprylic/capric glycerides and decaglycerol monocaprylate/caprate, less irritating to human skin [70]. Permeability experiments studying passage through a synthetic membrane made of silicone, as well as transdermal delivery to and through pig ear skin, showed a comparable efficiency for these newly-formulated MBGs and the parent microemulsions (permeability coefficients = 6 ± 1 × 10^−3^ and 6.3 ± 0.4 × 10^−3^ cm/h, respectively). Though the addition of a gelling agent improves the rheological behavior of the formulation for clinical use, previous studies by other groups have suggested that the addition of a gelling agent reduces transdermal delivery (by roughly 1.5-fold) for hydrophobic nonsteroidal anti-inflammatory drugs, highlighting a potential limitation of the formulation which would need more evaluation [71]. Compared to commercial microemulsion-based formulations such as Topicaine^®^ (ESBA Laboratories Inc., Jupiter, FL, USA, 30–60 mg/(cm^2^·h)), these lecithin-based formulations, with the same loading of 4% *w/w*, presented a much slower release profile (3 mg/cm^2^ in 18 h) for the local delivery of lidocaine, potentially beneficial for a longer-release formulation [72,73]. In addition to the reported works, studies of transdermal delivery in vivo and biocompatibility will be necessary to determine the clinical potential of this system.

Recently, the use of archaeosomes, liposome-like structures composed of archaeal lipids, have generated interest in drug delivery applications, with the Krishnan group at the National Research Council of Canada being a very active player in the field. Although most of their research is focused on the design of archaeosomes as immune adjuvants and delivery systems for parenteral administration, in 2017, Jia et al. investigated the transdermal permeability potentials of these structures. The authors screened the capacity of a pool of archaeosomes composed of archaeal total polar lipids, as well as semi-synthetic glycosylarchaeol, to diffuse through the skin and deliver ovalbumin (a common reference protein for vaccination experiments) and compared it to a standard DPPC/DPPG liposome formulation with similar size (100–300 nm) and comparable ovalbumin loading capacities. Using pig ear skin, the authors showed that all the tested particles generated from the total archaeal lipids had remarkably improved (up to 5 times) their capacity to cross the SC and deliver ovalbumin to the dermal layer compared to the liposomes composed of standard lipids or semi-synthetic glycosylarchaeol. While the authors observed that this improved permeability at least partially correlated with the fluid character of these archaeal vesicles, as well as with their negative surface charge, no other insights on the actual mechanism behind these activities has yet been provided [74]. While the small particle size is self-explanatory when it comes to permeation-enhancing properties of this system, a few previous works investigated similar systems for transdermal drug delivery applications and showed that their physical deformability is an important factor behind their significant SC permeability [75]. Nevertheless, further systematic structure-activity investigations will be needed to fully understand by which mechanism these compounds are able to cross the SC and promote the transdermal delivery of large hydrophilic molecules, which might lead to the design of ideal synthetic delivery methods. Aside from the mechanistic investigation, in vivo studies will be necessary to confirm the observed results and to assess the safety of such archaea-derived compounds.

**Figure 1:**
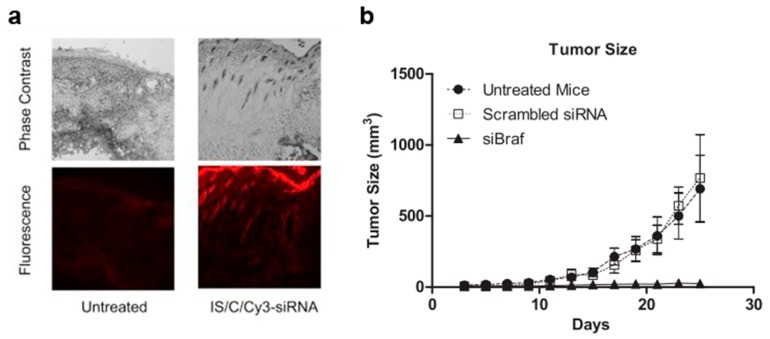
Carbon nanotube (CNT)-mediated delivery of siRNA in mice. (**a**) Representative images of tumor-bearing mouse skin treated with Cy3-labeled siRNA loaded CNTs; (**b**) tumor size evolution after topical administration of siRNA-loaded CNTs. Three days after the mice were injected with tumor cells, siRNA-loaded CNT solution was applied every 2 days for 25 days. Adapted with permission from [81]; published by Elsevier, 2014.

During the last decade, carbon nanotubes (CNTs) have gained popularity as potential drug and gene delivery vehicles for two main reasons: (1) their large inner volume, which allows the loading of either large pharmaceutical molecules, or larger quantities of smaller drugs, and (2) their observed capacity to operate as “nano-needles” which are able to effectively cross biological membranes, via a diffusion-like mechanism [76,77]. CNTs have thus been described for the delivery of several chemotherapeutic and antifungal agents such as cisplatin, doxorubicin, methotrexate, taxol, and amphotericin B, by parenteral administration [78]. However, their intrinsic hydrophobicity strongly limits their medical applications and consequently, different strategies have been described to overcome this limitation. Among them, surface decoration with polar or charged groups (such as the cationic polymer polyethylenimine) has been widely used to functionalize CNTs, leading to increased solubility and permitting the effective delivery of therapeutically active compounds [79]. Moreover, this functionalization with cationic residues has been shown to drastically improve the loading of nucleic acid-based therapeutics such as siRNA [80]. By virtue of their ability to cross biological membranes, a work from Western University in London, Ontario in 2014, investigated for the first time the use of single-walled carbon nanotubes (swCNTs) non-covalently functionalized with succinated polyethylenimine to topically deliver pharmaceutically active siRNA for the management of melanoma [81]. Functionalized swCNTs were loaded with an siRNA targeting Braf (a kinase involved in tumor growth via the MAPK pathway) at a remarkable *w*/*w* ratio of 2:1. These swCNTs demonstrated the selective downregulation of the targeted gene in melanoma cells (B16-F10), although no comparison with standard transfecting agents (i.e., cationic liposomes) was performed by the authors. Following these promising in vitro results, and based on a previous work demonstrating the ability of swCNTs to deliver low molecular-weight drugs transdermally, in association with an iontophoresis system [82]; the authors investigated the capacity of the particles to deliver siRNA across the epidermal layer, and to transfect melanoma cells in vivo after topical administration. Fluorescent microscopy of frozen sections of skin following the administration of swCNTs loaded with Cy3-labelled siRNA demonstrated the capacity of the nucleic acid sequence to cross the epidermis and reach the dermal layer (Figure 1a), while the same formulation without swCNTs did not cross the stratum corneum. Though these experiments highlighted the key role played by swCNTs in helping the siRNA to cross the epidermis, is should be noted that 10% DMSO, a well-known permeation enhancer, was added to the formulation. The authors then investigated the actual pharmaceutical potential of the swCNTs loaded with an anti-Braf siRNA in a mouse model of melanoma (intradermal inoculation of B16-F10 cells). Down-regulation experiments in the tumor cells indicated a remarkable 70% knockdown of Braf when delivering siRNA loaded in the functionalized swCNTs, 24 h after a single topical administration. Moreover, multiple administrations every 2 days for 25 days resulted in drastic inhibition of tumor growth (Figure 1b). Overall, these experiments by Siu et al. highlighted for the first time the potential of swCNTs to deliver pharmaceutics, even relatively large and hydrophilic molecules, across the epidermis, though the actual role of DMSO should be clarified. Nevertheless, prior to any actual clinical translation, the reported experiments will need to be validated in a more quantitative manner and in a more relevant animal model for transdermal studies, such as newborn pigs. Above all, while the described protocol used a functionalization step to increase the solubility of the CNTs, which has been reported to reduce their toxic accumulation and retention within the body, the actual fate of the CNTs as well as the non-degradable functionalization polymer will have to be carefully investigated [83,84].

## 5. Conclusions

In this brief review, we summarized and discussed the main topical and transdermal drug delivery technologies that have been developed in recent years in Canada. Overall, although the number of works describing new technologies appears limited at first glance, it is interesting to note that they cover a wide range of strategies, from nanotechnology to CPEs, to hollow MNs. Furthermore, the research spans from the development of innovative technologies based on new materials that have shown a remarkable capacity to enhance the transdermal delivery of high molecular weight drugs (e.g., functionalized CNTs), but require intensive and more quantitative studies to better identify their clinical potentials and biocompatibility profiles; to more fundamental studies with the broader goal of identifying superior enhancement compounds based on well-known materials. Finally, advanced strategies have been developed over the past decade, and are currently being further studied by private companies in clinical settings (e.g., the BiPhasix^TM^ technology) and could ideally soon reach the market, highlighting the favorable environment for the development of new medical and pharmaceutical technologies in the country.

Nonetheless, it should be noted that some transdermal drug delivery technologies undergoing rapid development are not currently represented in Canada. A notable example of this can be seen in polymeric MNs, two varieties of which have seen increasing use for drug delivery. The first of these are dissolving MNs made of soluble polymers, where a drug is loaded within the soluble polymeric matrix of the MNs, allowing release across the skin upon dissolution of the tips following application [85]. The Prausnitz group at Georgia Tech helped pioneer this technology in the context of non-invasive vaccination and drug delivery [86,87,88] and continue to work on this topic [89]. While research is also active globally, particularly in the context of delivering peptide-based drugs and macromolecules [90,91,92], no Canadian groups are currently investigating this class of MNs.

The other class of polymeric MNs being studied for transdermal drug delivery is swellable hydrogel MNs, typically made of crosslinked hydrophilic polymers able to swell by absorbing fluid from the skin. While these have also been used for sampling biological fluids from the skin [93], the Donnelly group at Queen’s University Belfast have primarily studied them for drug delivery purposes, as their swelling properties also allow drug molecules contained within the MNs to flow into the skin after application [94,95]. This research appears to have progressed significantly, with recent studies focusing on optimizing the system for clinical applications [96,97,98]. Iontophoresis is another transdermal drug delivery strategy currently experiencing worldwide growth [99]. By passing an electrical current through the skin, this technique serves to enhance skin permeability, as well as allow positively charged compounds to be transported into the skin by the resulting electric field. Though various groups are developing iontophoresis-based transdermal delivery methods for both small molecule- and peptide-based drugs [100,101], the topic is seemingly not undergoing active research within Canada.

These methods, alongside the ones discussed previously, serve to reveal the current state of transdermal drug delivery technology worldwide. Though Canada has generated meaningful contributions within the past decade, it remains clear that many opportunities for further work exist, if groups within Canada wish to further the progression of this field.

## Figures and Tables

**Table 1:** Transdermal drug delivery systems covered in the review.

Category	Technology	In Vivo Evaluation	Clinical Trial	Pros	Cons	Ref.
**Chemical permeation enhancer (CPE)-based systems**	Biphasic vesicles	Yes (guinea pig)	Phase II	Sustained release Versatility (small and large molecules)	Control of the delivered dose	[15,16,17,18,19,20,21,22,23,24]
	Ionic liquids (ILs) and active pharmaceutical ingredient-ionic liquids (API-ILs)	Yes (rats)	N/A	APIs with enhanced skin permeation properties of ionic liquids Properties can be fine-tuned	Requires specific choice of counter-ions Limited to small molecules	[42,43,47]
**Physical enhancer-based systems**	Hollow microneedles	Ex vivo: rabbit ear skin	Completed	Large doses Versatility	Manufacturing cost Potential clogging Skilled personnel	[53,54]
**Nanoparticle-based systems**	Lecithin-based microemulsions	N/A	N/A	Low skin irritation Sustained release and higher permeation compared to standard emulsions	Lecithin could lead to skin permeation complications	[61,64,65,69]
	Archaeosomes	N/A	N/A	Versatility Sustained release	Biocompatibility unclear Permeation mechanism unclear	[74]
	Carbon nanotubes (CNTs)	Yes (mice)	N/A	Effective skin permeation without CPEs High drug loading	Complexity Biocompatibility unclear	[81]
